# Essential oil composition variability among natural populations of *Pinus mugo* Turra in Kosovo

**DOI:** 10.1186/s40064-015-1611-5

**Published:** 2015-12-30

**Authors:** Avni Hajdari, Behxhet Mustafa, Gresa Ahmeti, Bledar Pulaj, Brigitte Lukas, Alban Ibraliu, Gjoshe Stefkov, Cassandra L. Quave, Johannes Novak

**Affiliations:** Department of Biology, Faculty of Mathematical and Natural Science, University of Prishtina, Mother Theresa St, 10000 Prishtinë, Kosovo; Institute of Biological and Environmental Research, Faculty of Mathematical and Natural Science, University of Prishtina, Mother Theresa St, 10000 Prishtinë, Kosovo; Institute of Animal Nutrition and Functional Plant Compounds, University of Veterinary Medicine, Veterinärplatz, 11210 Vienna, Austria; Department of Crop Production, Faculty of Agriculture and Environment, Agricultural University of Tirana, Kodër Kamëz, Tirana, Albania; Institute of Pharmacognosy, Faculty of Pharmacy, Ss. Cyril and Methodius University, Vodnjanska 17, 1000 Skopje, Republic of Macedonia; Center for the Study of Human Health, Emory University, 550 Asbury Circle, Candler Library 107E, Atlanta, GA 30322 USA; Department of Dermatology, Emory University School of Medicine, 1518 Clifton Road NE, CNR 5035, Atlanta, GA 30322 USA

**Keywords:** *Pinus mugo*, Essential oil, Natural variability, Kosovo

## Abstract

*Pinus mugo* Turra, is a native pine species in central and southern Europe, growing in high mountains area (altitudes 1.800–2.300 m.a.s.l.). In Kosovo, it is one of the native pines too, distributed in high altitudes in the Sharri Mountains and Albanian Alps Mountains. Its populations represent an important wealth of essential oil resources available, which make this species very important in terms of economic values. The chemical composition and yields of the essential oils of dwarf pine (*Pinus mugo* Turra) needles, twigs and cones from six wild populations in Kosovo were investigated with the aim to assess their natural variability. The identity of *P. mugo* was confirmed by morphology and DNA barcoding. Sixty-two compounds were identified representing 69–95 % of the total identified compounds. The yield ranged from 0.3–0.8 % v/w in needles, 1.0–2.4 % v/w in twigs and 0.1–0.5 % v/w in cones, depending on the origin of plant material and plant organs. α-Pinene (needles: 16.9–24.5 %; twigs: 4.5–8.8 %; cones: 3.1–5.6 %), β-pinene (needles: 1.5–5.4 %; twigs: 2.2–15.4 %; cones: 1.3–14.2 %), δ-3-carene (needles: 15.4–27.8 %; twigs: 24.0–51.6 %; cones: 10.5–31.5 %), limonene + β-phellandrene (needles: 1.9–5.9 %; twigs: 12.6–24.2 %; cones: 2.1–9.3 %), (*E*)-caryophyllene (needles: 4.4–8.9 %; twigs: 4.0–10.8 %; cones: 10.3–26.9 %) and germacrene D (needles: 4.0–8.3 %; twigs: 0.2–6.19 %; cones: 0.1–12.4 %) were the major components of the essential oil. Principal component analysis (PCA) and hierarchical cluster analyses (HCA) suggests that the population of *P. mugo* clustering is not related to their geographic location, but rather seemed to be linked to local selective forces acting on chemotype diversity. Low variability related to their geographic location has an economic importance since samples originating from different locations in Kosovo can treated with same standards.

## Background

*Pinus mugo* Turra, Pinaceae (Dwarf pine, Mountain pine) is a pine species that grows in the form of shrubs, up to 3.5 m high. It grows in high altitudes in the mountains of central and southern Europe at 1.800–2.300 m.a.s.l. (Tutin et al. 1964; Critchfield and Little 1966). Due to its wide ecological amplitude concerning environment factors, it is a pioneer species favourable to colonizing the areas inaccessible to other woody plants (Piękoś-Mirkowa H 1996). In Kosovo, it is one of the native pines distributed in high altitudes too, found in the Sharri Mountains and Albanian Alps Mountains in calcareous and dolomite substrate and its populations represent an important wealth of essential oil resources available, which make this species very important in terms of economic values.

In folk medicine *P. mugo* has been documented for use in various medicinal contexts, such as for wound healing (Redzić 2007), as an antitussive (Idolo et al. 2010), rheumatic, pulmonary diseases, antiseptic, anti-inflammatory, expectorant, and fluidizing properties (Venditti et al. 2013). *P. mugo* has a rich and diverse composition of secondary metabolites with different biological activities including antioxidative properties (Grassmann et al. 2003, 2005), secretolytic and antimicrobial effects (Ciuman 2012).

Several studies have reported the chemical composition of the *P. mugo* essential oil originating fromdifferent regions of the world (Kartnig et al.^1996^; Lawrence 1996; Kartnig et al. 1997; Tsitsimpikou et al. 2001; Ochocka et al. 2002; Venditti et al. 2013; Karapandzova et al. 2011; Stevanovic et al. 2005). Variation in the content and chemical composition between populations and plant organs (needles, twigs and cones) were reported as well (Kartnig et al. 1996, 1997).

According to ISO 9909 (2003), the following range (%) of main constituents is required in order to evaluate the quality of *Pinus mugo* essential oil: α-pinene (10–30 %), β-pinene (3–14 %), δ-3-carene (5–25 %), *p*-cymene (trace-2,5 %), limonene (8–14 %), terpinolene (1–8 %), bornyl acetate (1–5 %), (*E*)-caryophyllene (0.5–5 %), myrcene (3–11 %) and β-phellandrene (8–17 %).

Therefore, the principal aims of our study were to analyse the chemical composition of essential oils obtained from needles, twigs and cones of this plant and to assess the natural variation of essential oils between wild populations of *P. mugo* growing in Kosovo using a statistical approach with PCA and HCA.

## Results

The ITS (Internal Trascribed Spacer) sequences of the 18 *P. mugo* individuals exhibited identical sequence composition. No sequences of *P. mugo* were available in the public sequence database GenBank and sequences *of P. hwangshanensis, P. taiwanensis, P. luchuensis and P. densiflora* were identified as the closest relatives of our ITS sequences. *Pinus mugo* is a highly polymorphic taxon, it is usually divided into two subspecies: *Pinus mugo* subsp. *mugo* Turra (dwarf mountain pine) and *Pinus mugo* subsp. *uncinata* (Ramond) Domin (mountain pine) (Christensen 1987; Monteleone et al. 2006; Bogunić et al. 2011). Our analysed samples, based on their morphological characteristic (several curved trunks, long branches with base lies on the ground, while the end is erect) belong to *Pinus mugo* subsp. *mugo.* The occurrence of this subspecies in Balkans previously were recorded too (Alexandrov et al. 2011).

The results of the essential oil analysis extracted from *P. mugo* needles, twigs and cones essential oils collected from six locations in Kosovo are presented in Table 1. In total sixty-eight components were separated, which are listed in order of their elution from an HP-5MS column. Of these, sixty-two components were identified, which comprised 69–95 % of the total composition of the oils (Table 1). Hydrodistillation of the *P. mugo* needles, twigs and cones yielded light-yellowish essential oils. The yield of essential oils differed among the plant organs and population origin. The highest essential oil content was obtained from twigs (1.0–2.4 % v/w per dry weight) followed by needles (0.3–0.8 % v/w) and cones (0.1–0.5 % v/w) (Table 1).

**Table 1:** Composition (%) of the needles, twigs and cones oils of *Pinus mugo* from different locations

No.	Constituents^b^	“Sharri” National Park										“Bjëshket e Nemuna” National Park							
		Ostrovicë				Pashallarë			Oshlak			Hajle			Liqenat		Koprivnik		
		KI^a^	N	T	C	N	T	C	N	T	C	N	T	C	N	T	N	T	C
1	Tricyclene	926	0.7	tr	tr	0.7	tr	tr	0.9	0.2	tr	0.9	0.2	tr	1.1	0.2	0.9	tr	tr
*2*	*α-thujene*	*930*	*2.0*	*0.3*	*0.2*	*1.3*	*0.3*	*tr*	*1.3*	*0.2*	*tr*	*tr*	*0.2*	*tr*	*0.2*	*0.2*	*1.0*	*tr*	*0.2*
*3*	*α-pinene*	*940*	*22.6*	*7.9*	*3.1*	*17.2*	*9.7*	*4.0*	*17.0*	*8.8*	*5.4*	*19.9*	*5.6*	*4.6*	*23.9*	*7.4*	*24.5*	*6.9*	*5.7*
*4*	*Camphene*	*948*	*2.3*	*0.2*	*0.3*	*2.2*	*0.5*	*0.7*	*3.2*	*0.9*	*0.4*	*3.2*	*0.6*	*0.2*	*4.0*	*0.8*	*3.2*	*0.5*	*0.4*
*5*	*Thuja-2,4(10)-diene*	*960*	*tr*	*0.8*	*0.7*	*0.2*	*1.1*	*0.5*	*tr*	*0.7*	*0.7*	*0.3*	*0.5*	*2.0*	*tr*	*0.4*	*tr*	*0.3*	*0.3*
*6*	*Sabinene*	*975*	*0.9*	*1.3*	*0.4*	*0.8*	*1.1*	*0.4*	*1.0*	*2.2*	*0.5*	*0.9*	*1.7*	*0.5*	*0.8*	*1.3*	*1.0*	*1.0*	*0.3*
*7*	*β-pinene*	*979*	*1.6*	*5.5*	*1.6*	*3.7*	*14.4*	*3.1*	*5.5*	*15.4*	*14.2*	*2.8*	*2.2*	*1.6*	*3.4*	*3.5*	*2.7*	*2.8*	*1.4*
*8*	*Myrcene*	*990*	*1.4*	*2.1*	*1.1*	*1.8*	*1.6*	*1.6*	*2.6*	*2.3*	*1.4*	*2.4*	*2.7*	*1.1*	*6.1*	*10.3*	*3.2*	*4.3*	*4.6*
9	α-phellndrene	1005	tr	0.3	tr	0.2	0.3	tr	0.5	0.4	0.2	1.0	0.2	tr	0.3	0.3	0.3	0.3	tr
*10*	*δ-3-carene*	*1011*	*19.8*	*32.4*	*14.8*	*16.3*	*34.0*	*10.5*	*17.7*	*28.6*	*22.1*	*27.9*	*51.7*	*31.5*	*17.3*	*29.0*	*15.5*	*24.0*	*15.2*
11	α-terpinene	1018	0.4	0.2	0.2	0.3	0.2	tr	0.3	0.2	0.2	0.2	0.3	tr	0.2	0.2	0.3	0.2	tr
12	*p*-Cymene	1024	0.3	0.5	0.3	0.5	0.6	0.3	0.2	0.4	0.4	0.3	0.4	0.5	0.2	0.3	0.2	0.3	0.3
*13*	*Limonene* *+* *β-phellandrene*	*1031*	*2.0*	*24.3*	*2.2*	*3.3*	*12.7*	*2.0*	*5.9*	*17.9*	*9.3*	*3.8*	*14.3*	*4.0*	*2.3*	*21.1*	*4.8*	*23.2*	*6.3*
14	β-E-ocimene	1050	tr	tr	tr	0.5	tr	0.2	1.2	tr	0.2	0.8	0.6	0.2	0.7	0.2	0.8	tr	tr
15	γ-terpinen	1062	0.6	0.4	0.4	0.8	0.4	0.3	0.5	0.5	0.4	0.4	0.6	0.4	0.3	0.4	0.4	0.4	0.2
16	p-Mentha2,4(8)-diene	1088	tr	tr	tr	tr	tr	tr	tr	tr	tr	tr	0.2	tr	tr	tr	tr	tr	tr
*17*	*γ-terpinolene*	*1088*	*3.2*	*2.6*	*1.1*	*3.2*	*2.7*	*0.9*	*4.3*	*2.7*	*2.1*	*2.8*	*4.3*	*2.3*	*2.2*	*2.9*	*3.3*	*2.5*	*1.3*
*18*	*Linalool*	*1098*	*0.3*	*tr*	*3.6*	*0.3*	*tr*	*1.8*	*0.2*	*0.2*	*tr*	*tr*	*tr*	*tr*	*tr*	*tr*	*tr*	*tr*	*tr*
19	Unknown 1	1127	tr	0.4	0.2	0.2	0.5	0.4	tr	0.4	0.4	tr	tr	tr	tr	tr	tr	0.2	tr
20	*E*-pinocarveol	1139	0.2	tr	0.2	0.2	0.2	tr	tr	0.2	0.3	0.3	0.5	0.4	0.3	0.4	0.4	0.2	tr
21	Camphor	1146	0.4	0.3	0.3	0.5	0.3	0.5	0.2	0.4	tr	0.5	tr	tr	0.4	tr	0.4	tr	tr
22	Borneol	1165	0.7	1.0	1.0	0.5	0.3	0.5	0.5	0.4	0.2	0.3	0.5	0.4	0.4	0.5	0.5	0.5	0.2
23	Terpinene-4-ol	1177	tr	0.2	tr	tr	0.2	tr	tr	0.2	tr	tr	0.3	0.3	tr	0.2	tr	0.2	tr
24	Meta-cymen-8-ol	1179	tr	0.3	tr	0.2	0.3	tr	tr	0.2	tr	tr	0.3	0.2	tr	0.2	tr	tr	tr
25	*p*-cymen-8-ol	1183	tr	0.4	tr	tr	0.3	tr	tr	0.2	tr	tr	0.2	tr	tr	0.2	tr	0.3	0.4
26	α-terpinol	1188	0.3	0.2	0.8	0.3	0.2	0.3	0.2	0.3	tr	tr	tr	tr	0.2	tr	0.2	tr	tr
27	Methyl salicylate	1191	tr	tr	0.2	tr	tr	tr	tr	0.3	tr	tr	tr	tr	tr	0.2	tr	tr	tr
28	Myrtenol	1195	0.2	1.2	0.2	0.4	0.8	tr	0.2	0.7	0.5	0.2	0.6	0.2	0.2	0.8	0.2	0.9	0.2
29	Thymol methyl ether	1235	0.5	0.2	1.1	0.8	0.2	0.9	0.3	0.2	tr	tr	0.2	tr	tr	tr	0.2	tr	tr
30	Linalool acetate	1256	0.2	tr	1.2	0.2	tr	0.6	0.2	0.2	tr	0.3	tr	0.2	0.2	tr	0.2	tr	tr
*31*	*Bornyl acetate*	*1285*	*2.6*	*0.4*	*0.5*	*5.2*	*1.2*	*1.2*	*4.3*	*2.6*	*1.5*	*4.6*	*1.8*	*0.8*	*8.1*	*3.0*	*7.1*	*2.4*	*1.3*
32	β-terpinyl acetate	1352	0.2	tr	tr	0.2	tr	tr	0.2	tr	tr	0.3	tr	tr	0.2	tr	0.2	tr	tr
33	α-copane	1376	1.5	tr	0.2	tr	tr	0.2	tr	0.4	tr	tr	tr	tr	tr	tr	tr	tr	tr
34	β-Bourbonene	1384	0.2	0.3	tr	tr	tr	tr	tr	0.2	tr	0.2	0.3	tr	0.2	0.3	tr	0.2	tr
*35*	*β-elemene*	*1391*	*1.2*	*0.9*	*0.3*	*2.1*	*1.0*	*0.2*	*1.7*	*0.6*	*0.6*	*0.7*	*1.1*	*0.3*	*1.2*	*1.2*	*1.8*	*1.0*	*0.5*
36	Longifolene	1407	tr	0.4	1.4	0.5	0.4	2.8	tr	tr	tr	tr	tr	tr	tr	tr	tr	tr	tr
37	Unknown 2	1420	0.5	tr	0.9	0.2	tr	0.5	tr	tr	tr	tr	tr	tr	tr	tr	tr	tr	0.3
*38*	*E-caryophyllene*	*1418*	*9.0*	*7.3*	*19.2*	*8.1*	*6.1*	*20.5*	*5.3*	*5.9*	*10.4*	*4.5*	*4.0*	*16.6*	*5.1*	*6.7*	*4.6*	*10.9*	*27.0*
39	β-copaene	1432	0.3	tr	0.5	0.2	tr	1.1	0.2	tr	tr	0.3	tr	tr	0.3	tr	0.2	tr	tr
40	α-Humulene	1454	tr	0.5	tr	0.6	1.0	tr	0.6	0.7	2.6	0.5	tr	1.8	0.5	0.5	0.8	1.8	4.5
*41*	*Aromadendrene*	*1458*	*1.5*	*1.2*	*3.2*	*1.1*	*tr*	*3.3*	*0.3*	*0.3*	*tr*	*0.2*	*0.6*	*1.0*	*0.3*	*0.6*	*tr*	*tr*	*tr*
42	Cis-muurola-4(14),5 diene	1458	0.2	tr	0.2	0.2	tr	0.2	tr	tr	tr	tr	tr	tr	0.2	tr	0.2	tr	tr
43	β-chamigrene	1477	0.7	0.3	0.3	0.2	tr	tr	0.4	tr	0.5	0.2	0.2	0.7	0.5	0.5	0.2	0.3	1.2
44	γ-gurjunene	1477	tr	tr	tr	tr	tr	tr	tr	tr	tr	tr	tr	tr	tr	tr	tr	tr	tr
45	γ-muurolene	1479	0.4	tr	0.6	0.6	tr	1.1	0.4	tr	tr	0.2	tr	tr	0.3	tr	0.3	0.6	1.2
*46*	*Germacrene D*	*1481*	*5.5*	*0.4*	*1.2*	*5.6*	*0.6*	*3.0*	*9.9*	*0.4*	*0.2*	*4.0*	*tr*	*1.3*	*5.6*	*0.8*	*8.4*	*6.2*	*12.5*
47	β-selinene	1490	0.3	tr	tr	0.5	0.2	tr	0.2	tr	tr	0.2	tr	tr	0.3	tr	0.2	tr	tr
48	γ-amorphene	1495	0.2	tr	0.2	0.3	tr	0.4	0.2	tr	tr	tr	tr	tr	0.2	tr	0.2	0.2	0.4
*49*	*Bicyclogermacrene*	*1500*	*2.1*	*0.7*	*0.3*	*3.0*	*0.8*	*0.5*	*2.2*	*0.6*	*tr*	*3.4*	*0.6*	*tr*	*3.1*	*1.0*	*2.2*	*0.5*	*0.4*
50	α-muurolene	1500	1.0	0.2	0.3	0.6	0.2	0.3	0.6	tr	tr	0.3	tr	tr	0.4	tr	0.4	tr	0.2
*51*	*γ-cadinene*	*1519*	*2.2*	*0.2*	*0.3*	*1.2*	*0.2*	*0.6*	*0.7*	*0.2*	*tr*	*0.6*	*0.2*	*tr*	*0.7*	*0.4*	*0.8*	*0.4*	*0.9*
*52*	*δ-cadinene*	*1523*	*2.1*	*0.5*	*0.7*	*3.6*	*0.9*	*1.5*	*2.3*	*0.5*	*0.3*	*2.2*	*0.4*	*tr*	*2.3*	*0.8*	*2.2*	*0.9*	*1.8*
53	Trans-cadina-1,4-diene	1534	tr	tr	0.4	tr	tr	0.5	tr	tr	tr	0.3	tr	tr	tr	tr	tr	tr	0.3
54	α-cadinene	1538	0.6	tr	0.7	0.4	tr	0.4	0.5	tr	0.8	0.2	tr	1.0	tr	tr	0.2	tr	0.4
55	E-nerolidol	1561	0.4	tr	0.2	0.7	0.2	0.4	0.6	tr	tr	0.7	tr	tr	0.6	0.2	0.7	tr	tr
56	Unknown 3	1582	0.7	0.5	tr	0.9	0.6	tr	0.4	0.3	tr	0.8	0.4	tr	0.7	0.4	0.3	0.3	tr
57	Spathulenol	1578	1.2	0.3	0.2	1.4	0.4	0.3	1.1	0.2	tr	1.3	0.2	tr	1.3	0.2	1.2	tr	0.5
58	Cariophyllene oxide	1583	1.6	0.2	0.4	1.9	0.6	0.5	1.5	0.2	tr	1.8	0.3	tr	1.8	0.4	1.7	0.2	0.7
59	Juneol	1618	tr	tr	0.6	tr	tr	0.5	tr	tr	0.8	tr	tr	0.2	tr	tr	tr	tr	0.2
60	Epi-α-cubenol	1627	0.4	tr	tr	0.3	tr	tr	0.3	tr	tr	0.3	tr	tr	0.2	tr	0.2	tr	tr
61	Epi-α-cadinol	1640	1.6	0.2	0.3	0.7	0.3	0.4	0.7	0.2	0.4	0.7	tr	0.2	0.3	0.2	0.6	0.5	0.6
62	α-Muurolol	1645	tr	0.2	1.3	0.2	0.3	1.6	tr	0.2	2.6	tr	tr	3.0	tr	0.2	tr	0.5	1.3
63	Amorpha-4,9-dien-2-ol	1700	tr	tr	0.2	tr	tr	0.2	tr	tr	0.5	tr	tr	0.7	tr	tr	tr	tr	0.4
64	Manooloxide	1987	tr	tr	tr	tr	tr	0.3	0.2	tr	0.7	0.2	tr	1.1	0.2	tr	tr	0.2	0.9
65	Abietadiene	2087	tr	tr	0.2	tr	tr	0.3	tr	tr	1.1	tr	tr	1.1	tr	tr	tr	0.2	0.6
*66*	*Unknown 4*	*2116*	*tr*	*0.4*	*24.2*	*1.0*	*tr*	*19.1*	*tr*	*tr*	*8.5*	*tr*	*tr*	*0.5*	*tr*	*tr*	*tr*	*1.7*	*3.2*
*67*	*Unknown 5*	*2159*	*2.1*	*tr*	*tr*	*0.7*	*0.2*	*0.2*	*0.5*	*tr*	*0.8*	*0.5*	*tr*	*tr*	*0.2*	*tr*	*0.4*	*tr*	*0.2*
*68*	*Unknown 6*		*tr*	*0.6*	*5.5*	*0.8*	*0.8*	*7.1*	*0.3*	*0.7*	*8.2*	*0.8*	*0.4*	*19.6*	*0.2*	*1.0*	*tr*	*0.9*	*tr*
	Total identified		97.6	96.8	68.9	95.8	96.8	71.4	98.3	98.1	81.5	97.1	98.6	79.1	98.6	98.0	98.6	95.8	94.8
	Yield %v/w (min and max.values)		0.5–0.6	1.1–1.2	0.4–0.5	0.3–04	1.0–1.1	0.3–0.4	0.5–0.6	1.4–1.8	0.2–0.3	0.4–0.5	1.8–2.2	0.1–0.2	0.5–0.8	1.5–1.8	0.4–0.8	1.8–2.4	0.3–0.5
	Monoterpenes		57.2	79.0	26.2	53.1	79.7	24.9	62.1	80.9	57.5	67.2	86.1	50.1	63.2	78.2	62.3	67.0	36.3
	Oxygenatedmonoterpenes		2.20	4.10	6.50	2.60	3.10	4.00	1.50	3.50	1.80	1.70	2.60	1.80	1.80	2.50	1.90	2.50	1.40
	Sesquiterpenes		28.8	13.2	30.1	28.8	11.9	36.9	25.6	10.2	15.2	18.	7.6	19.8	21.1	13.2	23.0	23.0	51.7
	Oxygenated sesquiterpenes		5.3	1.2	3.2	5.3	2.0	3.9	4.3	1.0	4.4	4.9	0.7	4.2	4.3	1.2	4.1	1.6	3.9
	Diterpenes		0.1	0.0	0.3	0.1	0.0	0.6	0.2	0.0	1.8	0.2	0.0	2.2	0.2	0.1	0.1	0.4	1.5
	Other hydrocarbons		3.6	0.8	3.0	6.5	1.6	2.	5.0	3.3	1.6	5.4	2.2	1.1	8.2	3.3	7.70	2.5	1.3
	Unidentified		2.8	1.7	30.7	3.6	1.7	26.9	1.3	1.1	17.7	2.3	0.8	20.8	1.2	1.5	0.9	3.0	3.9

The percentage for each population represents the mean values a of n calculated samples (n = 2–4 samples). Italics marked compounds were chosen for HCA and PCA statistical analyses, tr = trace < 0.1 %

*N* needles, *T* twigs, *C* cones

^a^Kovats indices calculated against a C9–C22 n-alkanes mixture on the HP5 MS column

^b^Compounds are listed in order of elution from a HP-5MS column and their percentages were obtained by FID peak-area normalisation

The main compounds differed among plant organs and plant population too. In needles, the major components were: α-pinene (17.0–24.5 %), followed by δ-3-carene (15.5–27.9 %), germacrene D (4.0–9.9 %) and (*E*)-caryophyllene (4.3–9.0 %). In twigs the major components were: 3-δ-carene (24.0–51.7 %) followed by limonene + β-phellandrene (12.7–24.3 %), (*E*)-caryophyllene (4.0–10.9 %), β-pinene (2.2–15.4 %) and α-pinene (4.5–8.8 %), whereas in cones the major components were δ-3-carene (10.5–31.5 %), followed by (*E*)-caryophyllene (10.4–27.0 %), an unknown compound (0.0–24.2 %), β-pinene (1.4–14.4 %) and germacrene D (0.1–12.4 %). Concentrations of these constituents also differed depending on the origin of the plant population (Table 1).

Monoterpenes constituted the highest percentage of all components (24.9–86.1 %), followed by sesquiterpenes (7.6–51.7 %), oxygenated sesquiterpenes (1.0–6.5 %), oxygenated monoterpenes (1.4–6.6 %), diterpenes (0.00–2.2 %), other hydrocarbons (0.3–8.2 %) and an unknown compound (0.8–30.7 %).

Hierarchical cluster analysis (HCA) and principal component analyses (PCA) were used as statistical tools in order to identify possible relationships between volatile compounds obtained from plant organs (needles, twigs and cones) and geographical location of the plant populations. For statistical analyses the oil components with concentrations higher than 2 % (italic in Table 1) of the total oil were selected.

The analysis of variance showed that the means for the majority of the oil compounds differed significantly (*p* > 0.05) among plant organs but not between populations (Table 1). Nevertheless, both the interaction between population location and plant organs was found to be statistically significant in respect to the chemical composition of *P. mugo* essential oil (*p* > 0.05).

**Fig. 1:**
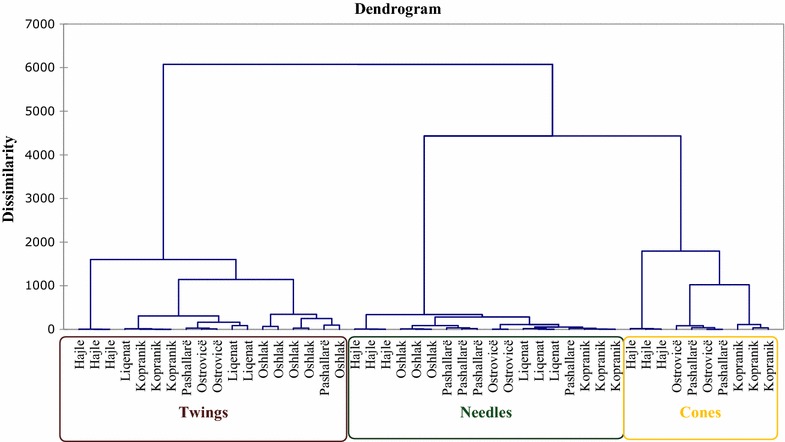
Two-dimensional dendrogram obtained by the cluster analysis of the essential oils of six populations of *Pinus mugo* based on the unweighed pair-group method (square Euclidean distance)

The general structure of the dendrogram generated by HCA indicated the existence of three main clusters, corresponding with the chemical composition of plant organs (Fig. 1). The first cluster includes the oils obtained from twigs, the second cluster groups oils obtained from needles, whereas the third cluster groups oils obtained from cones. HCA identified the closest connection as being between needles and cones; twigs were the most distant group (Fig. 1).

**Fig. 2:**
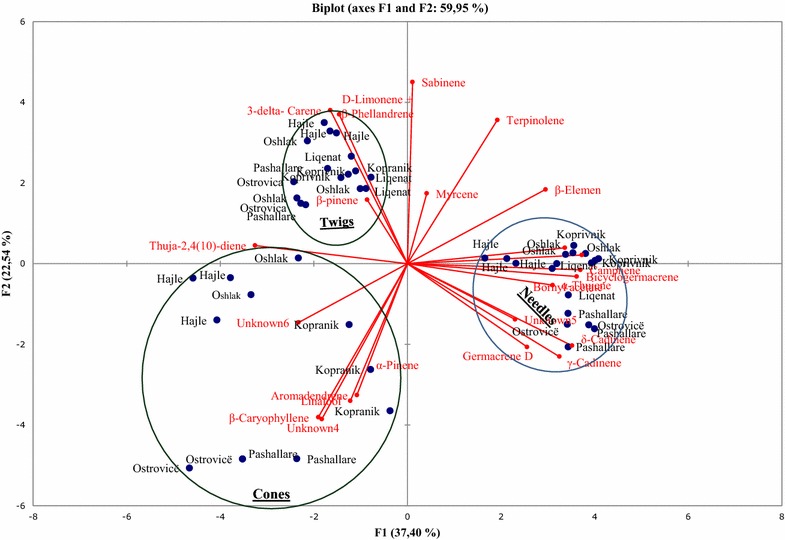
Principal component analysis of the oil constituents obtained from needles, twigs and cones of six populations of *P. mugo*

PCA confirmed this clustering by HCA; the two-dimensional axial system of the PCA identified three groups too. Based on the chemical composition of their essential oils, samples were grouped regarding the plant organs from which oils were obtained (Fig. 2). Camphene, α-thujene, bicyclogermacrene, bornyl acetate, δ-cadinene, γ-cadinene and germacrene D were the principal components that contributed to clustering of samples obtained from needles. δ-3-carene, sabinene, limonene + β-phellandrene, β-pinene were the primary components that contributed to the clustering of the oils obtained from twigs, whereas (*E*)-caryophyllene, aromadendrene, linalool, α-pinene, unknown 4 and unknown 6 were the primary components that contributed to the clustering of the oils obtained from cones. PCA results showed that the first two principal axes represented 60 % of the total variance, thus, the first axis contributed with 37 % of the total variation whereas the second axis with 23 % (Fig. 2).

With the aim to see the variability between populations PCA was performed separately for samples obtained from needles, twigs and cones.

**Fig. 3:**
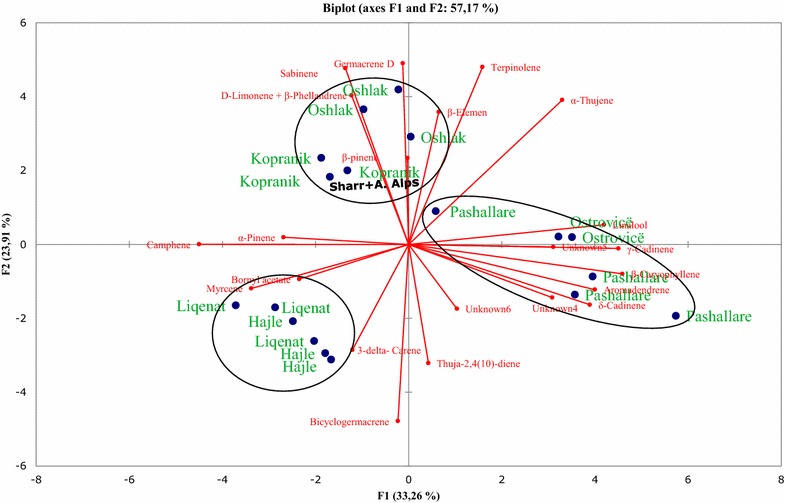
Principal component analysis of the oil constituents obtained from needles of six populations of *P. mugo*

**Fig. 4:**
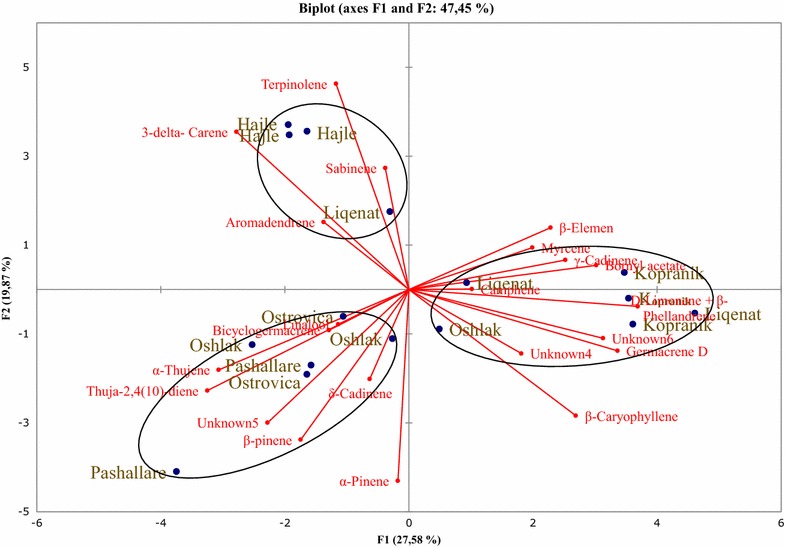
Principal component analysis of the oil constituents obtained from twigs of six populations of *P. mugo*

**Fig. 5:**
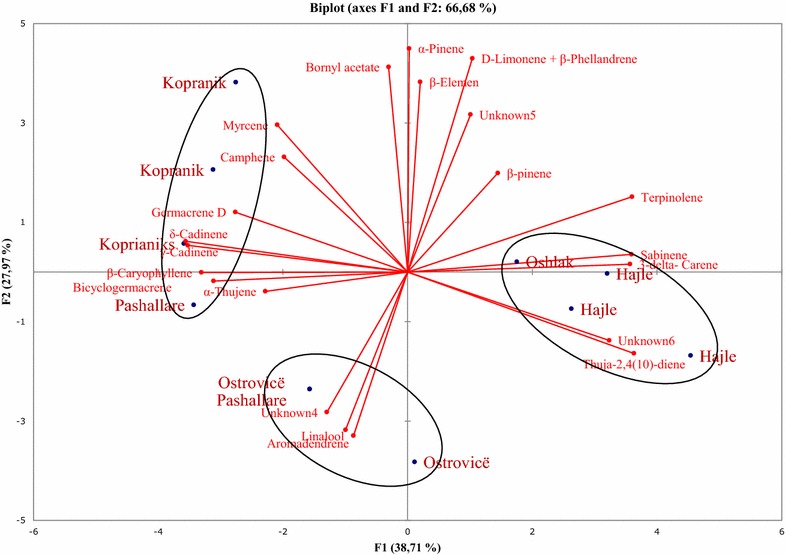
Principal component analysis of the oil constituents obtained from cones of six populations of *P. mugo*

The two-dimensional axial system of the PCA suggests the existence of three groups of essential oils obtained from needles of *P. mugo* (Fig. 3). Thus, limonene + β-phellandrene, sabinene, germacrene D, β-pinene and β-elemene were the principal components that contributed to population clustering of the plants from Oshlak and Kopranik/Koprivnik. α-Thujene, linalool, unknown no. 5, γ-cadinene, (E)-caryophyllene, aromadendrene, unknown no. 4, δ-cadinene were the primary components that contributed to the clustering of the population from Pashallarë and Ostrovicë. The population from Liqenat and Hajle were dominated by bornyl acetate, myrcene and δ-3-carene (Fig. 3). PCA results obtained from twigs identified three groups of populations too, based on the chemical composition of their essential oils (Fig. 4). Terpinolene, δ-3-carene, sabinene and aromadendrene were the principal components that contributed to population clustering of the plants from Hajle and Liqenat. Bicyclogermacrene, linalool, α-thujene, thuja-2,4(10)-diene, δ-cadinene, unknown 5, and β-pinene were the primary components that contributed to the clustering of the population from Ostrovicë, Oshlak and Pashallarë, whereas the population from Liqenat, Kopranik/Koprivnik and Oshlak were dominated by β-elemene, myrcene, γ-cadinene, bornyl acetate, limonene + β-phellandrene, unknown no. 6, germacrene D, unknown no. 4, (*E*)-caryophyllene (Fig. 4). The PCA results obtained from essential oil composition of cones identified three groups of populations based on the chemical composition of their essential oils (Fig. 5). Myrcene, camphene, germacrene D, δ-cadinene, γ-cadinene, (*E*)-caryophyllene, bicyclogermacrene and α-thujene were the principal components that contributed to population clustering of the plants from Kopranik/Koprivnik and a location in the site of Pashallar. Unknown no. 4, linalool and aromadendrene were the primary components that contributed to the clustering of the population from Ostrovicë, and Pashallarë. The population from Hajle and Oshlak were dominated by terpinolene, δ-3-carene, sabinene, unknown 6 and thuja-2,4(10)-diene (Fig. 5).

## Discussion

To characterize the natural variability between *P. mugo* populations, we compared the yield and chemical composition of the essential oil collected from six locations in Kosovo. The essential oil yields of our populations differed depending on the population origins and ranged from 1.0–2.4 % v/w weight in twigs followed by needles (0.3–0.8 % v/w) and cones (0.1–0.5 % v/w) based on dry weight. In general, the highest yield of essential oils was found in populations originating from “Bjeshkët e Nemuna” National Park. Previous work has shown that the yield of *P. mugo* needles varied from 0.15 to 0.65 % (Karapandzova et al. 2011).

In all of the Kosovar populations, the primary components in needles were α-pinene followed by δ-3-carene, germacrene and (*E*)-caryophyllene. δ-3-carene followed by limonene + β-phellandrene, (*E*)-caryophyllene, β-pinene, α-pinene in twigs, whereas δ-3-carene, followed by (*E*)-caryophyllene, unknown no. 4, β-pinene, germacrene D were main constituents in cones; similarly, α-pinene, δ-3-carene, (*E*)-caryophyllene were also the primary components of needles essential oils in populations from Macedonia (Karapandzova et al. 2011), δ-3-carene, α-pinene, β-pinene, and β-phellandrene were main constituent of oils obtained from needles and branches of *P. mugo* originated from “Sharri” National Park (Stevanovic et al. 2005), and from Austria (Kartnig et al. 1996, 1997). The essential oil of needles originating from Edinburgh (UK), on the other hand, were reported to contain α-pinene, γ-cadinene, caryophyllene oxide, sandaracopimara-8 (14),15-diene and α-cadinol as main constituents (Tsitsimpikou et al. 2001). Unlike our results, the main constituents of the essential oil originating from Majella National Park (Italy) were bornyl acetate, a-terpineol (*E*)-caryophyllene α -cadinol, and etc. (Venditti et al. 2013). Previously, variations in *P. mugo* essential oil composition was reported for samples originating from different populations in Austria (Kartnig et al. 1996, 1997).

The quantitative composition of the essential oil obtained from needles and twigs together, with few exceptions, meets the required standards of ISO 9909 (2003), whereas essential oil obtained separately from needles and twigs do not meet ISO 9909 standards (2003) (Table 2).

**Table 2:** The major constituents (%) of the essential oil of *Pinus mugo* in comparison with ISO 21093 (2003)

Constituents	“Sharri” National Park										“Bjëshkete Nemuna” National Park									ISO 21093
	Ostrovicë				Pashallarë			Oshlak			Hajle			Liqenat			Koprivnik			
	KI	N	T	N + T	N	T	N + T	N	B	N + T	N	T	N + T	N	T	N + T	N	T	N + T	
α-pinene	940	22.6	7.9	*15.25*	17.2	9.7	*13.45*	17	8.8	*12.9*	19.9	5.6	*12.75*	23.9	7.4	*15.65*	24.5	6.9	*15.7*	*10–30*
β-pinene	979	1.6	5.5	*3.55*	3.7	14.4	*9.05*	5.5	15.4	*10.45*	2.8	2.2	*2.5*	3.4	3.5	*3.45*	2.7	2.8	*2.75*	*3–14*
Myrcene	990	1.4	2.1	*1.75*	1.8	1.6	*1.7*	2.6	2.3	*2.45*	2.4	2.7	*2.55*	6.1	10.3	*8.2*	3.2	4.3	*3.75*	*3–11*
δ-3-CARENE	1011	19.8	32.4	*26.1*	16.3	34	*25.15*	17.7	28.6	*23.15*	27.9	51.7	*39.8*	17.3	29	*23.15*	15.5	24	*19.75*	*5–25*
p-Cymene	1024	0.3	0.5	*0.4*	0.5	0.6	*0.55*	0.2	0.4	*0.3*	0.3	0.4	*0.35*	0.2	0.3	*0.25*	0.2	0.3	*0.25*	*tr-2.5*
Limonene + β-Phellandrene	1031	2	24.3	*13.15*	3.3	12.7	*8*	5.9	17.9	*11.9*	3.8	14.3	*9.05*	2.3	21.1	*11.7*	4.8	23.2	*14*	*Lim. 8–14* *β-Phell. 8-17*
Terpinolene	1088	3.2	2.6	*2.9*	3.2	2.7	*2.95*	4.3	2.7	*3.5*	2.8	4.3	*3.55*	2.2	2.9	*2.55*	3.3	2.5	*2.9*	*1–8*
Bornyl acetate	1285	2.6	0.4	*1.5*	5.2	1.2	*3.2*	4.3	2.6	*3.45*	4.6	1.8	*3.2*	8.1	3	*5.55*	7.1	2.4	*4.75*	*1–5*
*E*-caryophyllene	1418	9	7.3	*8.15*	8.1	6.1	*7.1*	5.3	5.9	*5.6*	4.5	4	*4.25*	5.1	6.7	*5.9*	4.6	10.9	*7.75*	*0.5–5*

Italics marked symbols represents the percentages of each compounds obtained by distillation of twigs and leaves together, as well as the International Standards (ISO 21093) requirements for essential oils obtained from branches of *P. mugo*

ISO 21093-minimum and and maximum of constituents given regarding  % ISO 21093

*N* needles, *T* twigs, *N* *+* *T* needles and twigs

The differences between plant populations were not as high as were the differences between plant organs. This is not surprising because different plant organs show a completely different gene expression profile adapted to the function of the respective organ. Small differences between the populations tested possibly indicate a high genetic relationship among the populations. The significant interaction between populations and plant organs, however, are probably an indication for an environmental influence on gene expression profiles.

PCA and HCA statistical analyses indicated the existence of three main clusters, corresponding to chemical composition of the plant organs, demonstrating that the biggest differences in essential oil chemical composition were found between plant organs. Such variation in chemical composition of the essential oil obtained from different plant organs were previously reported by (Kartnig et al. 1996, 1997). Statistical analyses also identified three main groups of populations, based on the chemical composition of the essential oils obtained separately from needles, twigs and cones. The plot established according to the first two PCA axes indicates the existence of three groups of essential oils obtained from needles *P. mugo*. The first group includes populations originating from Oshlak and Kopranik/Koprivnik (“Shari” and “Bjeshkët e Nemuna” National Park). The second group from Pashallarë and Ostrovicë (“Shari” National Park) and third from Liqenat and Hajlë (“Bjeshkët e Nemuna” National Park). Regarding the chemical composition of the essential oils obtained from twigs, PCA identified three groups of populations too; the first group included populations originating from Hajle and Liqenat (Bjeshkët e Nemuna” National Park), the second from Ostrovicë, Oshlak and Pashallarë (“Shari” National Park), and the third from Liqenat, Kopranik/Koprivnik and Oshlak (“Shari” and “Bjeshkët e Nemuna” National Park). PCA analysis of the cone essential oil composition also resulted in identification of three groups of populations as well: the first group includes populations originating from Kopranik/Koprivnik and a location in the site of Pashallar (“Shari” and “Bjeshkët e Nemuna” National Park), the second from Ostrovicë, and Pashallarë (“Shari” National Park), and the third from Hajle and Oshlak (“Shari” and “Bjeshkët e Nemuna” National Park).

## Conclusions

The yield and chemical composition of the essential oil differed depending on population origins and plant organs, thus the primary components in needles were α-pinene followed by δ-3-carene, germacrene and (*E*)-caryophyllene. δ-3-carene followed by limonene + β-phellandrene, (*E*)-caryophyllene, β-pinene, α-pinene in twigs, whereas δ-3-carene, followed by (*E*)-caryophyllene, unknown no. 4, β-pinene, germacrene D. HCA and PCA statistical analyses confirm the differences in chemical composition depending on the plant organs and the geographical origin of the plant populations. Thus, the samples used in this study were collected in two Kosovar National Parks (“Shari” and “Bjeshkët e Nemuna” National Park), which are separate from one another and located approximately 100 km apart. Due to geographic isolation of the mountainous areas and anemophilous pollination of the *P. mugo,* we expected to find two distinct groups based on their chemical composition of essential oils. Statistical analyses of our results did not support this hypothesis, however, as some of our samples from “Shari” and “Bjeshkët e Nemuna” National Park were grouped together. This uniformity can be explained by past distributions of this species, which was more widespread and without interruption. The current areal is a contraction of the past areal due to the climatic changes following the last ice age. Realistically, the two studied populations are a residue of a wider and older population and it is not surprising to observe similarities in their respective compositions since this separation has occurred in recent times.

The spatial distribution of the populations suggests that their clustering is not related to their geographic location, but rather seemed to be linked to local selective forces acting on chemotype diversity. Low variability related to their geographic location has an economic importance since samples originating from different locations in Kosovo can treated with same standards.

## Methods

### Plant material

**Fig. 6:**
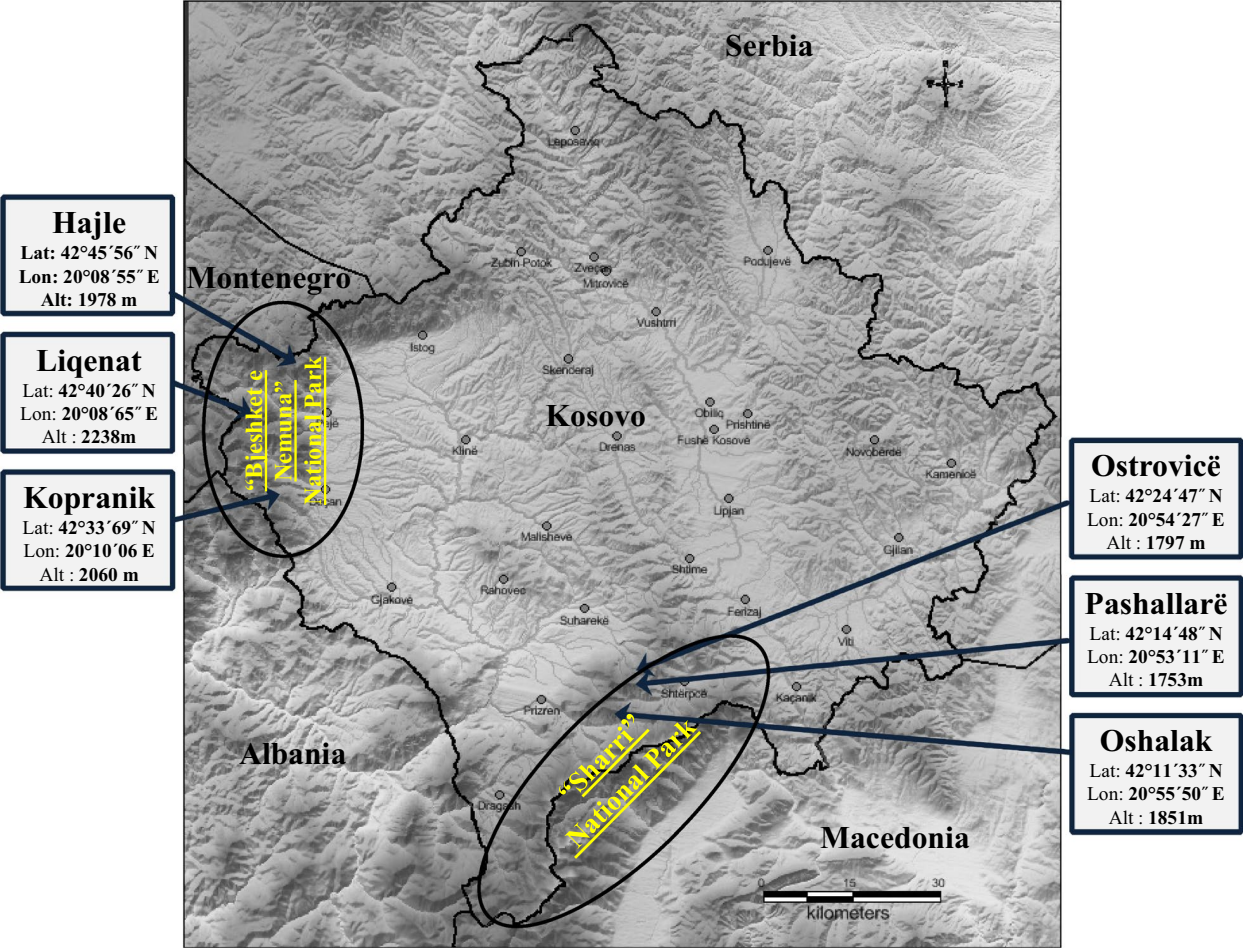
Basic characterization of the sites from where the plant materials of *P. mugo* were collected

Plant material of *Pinus mugo* was collected from July to September 2013 from six different wild populations in Kosovo. Three of the populations originated from “Sharri” National Park, whereas three others from “Bjeshkët e Nemuna” National Park (Fig. 6). The collection sites were recorded using a Global Positioning System (GPS) receiver (GARMIN, eTrex^®^ 30). Two to four replicate samples of needles, twigs and cones were analysed, each sample was gathered from 2–3 individual plants from each population. Samples were distilled and analysed separately. Voucher specimens of each population were deposited to the Herbarium of the Department of Biology, University of Prishtina.

### Essential oil extraction

Plant material was air-dried in shade at room temperature and cut into small pieces (<0.5 cm). Separated needles, twigs (only wooden parts) and cones were subjected to essential oil distillation. For distillation, 50 g of dry tissue was placed into 0.5 L of water in a 1 L flask and distilled at a rate of 3 mL/min in a Clevenger apparatus for 3 h. The samples were stored in the dark at −18 °C in the freezer pending further analysis. The yield of essential oil is expressed as a percentage of the mass of the essential oil with respect to the air-dried material (% v/w of dried material).

### DNA barcoding

Nuclear ITS (internal transcribed spacer) of three individual plants of each of the six populations was sequenced to confirm species identity and to proof whether intra-specific DNA polymorphisms are present.

Genomic DNA was extracted from dried needles using a modified CTAB protocol (Doyle 1991). The internal transcribed spacer (ITS) region of nuclear DNA, including ITS1, ITS2 and the 5.8S gene, was amplified and sequenced with the primers PIN2451F and PIN26S25R (Nickrent et al. 1994; Liston et al. 1999). For a 15 μL PCR reaction, 1 μL of genomic DNA solution (1:50 dilution of the original DNA extract) was added to a master mix containing 1 × PCR buffer B, 2.5 mM MgCl_2_, 133 μM dNTPs, 0.6 U HotFire Taq polymerase (all reagents from Solis BioDyne, Tartu, Estonia) and 0.6 μM forward and reverse primer (Invitrogen, Lofer, Austria). The PCR cycle profile included an initial denaturation step at 95 °C for 15 min, followed by 35 cycles at 95 °C for 45 s, at 55 °C for 45 s, and a final elongation step at 72 °C for 90 s. The PCR products were purified with the enzymes ExoI and SAP (Fermentas, Burlington, Canada) and were sequenced by an external company (Ibl, Gerasdorf, Austria). The obtained sequences of the samples were edited, assembled and aligned with Geneious 5.3.4. (Biomatters Ltd., Auckland, New Zealand). All were deposited in GenBank (accession numbers in GenBank: KR052968- KR052986).

### GC and GC–MS analyses

GC/FID analyses were performed using an Agilent 7890A GC System equipped with an FID detector (Agilent Technologies). The separation was conducted on a HP-5MS column 30 m × 0.25 mm with 0.25 μm film thickness. Helium was used as carrier gas with an initial flow rate of 0.6 mL/min and subsequently at a constant pressure of 50.0 psi. The front inlet was maintained at 250 °C in a split ratio of 50:1. The GC oven temperature increased from 60 to 260 °C at a rate of 5 °C/min and the FID operated at 250 °C with an air flow of 350 mL/min and a hydrogen flow of 35 mL/min. The injection volume was 1.0 μL.

GC/MS analyses were performed using an Agilent 7890A GC system coupled to a 5975C MSD (Agilent Technologies). The ionisation energy was 70 eV with a mass range of 40–400 m/z. The separation was conducted with the same column and temperature program as for the analytical GC.

Identification of the essential oil components was made by comparing their Kovats retention indexes with those in literature (Adams 2009). The calculation of the Kovats index was made based on a linear interpolation of the retention time of the homologous series of *n*-alkanes (C9–C22) under the same operating conditions. The components were also identified by comparing the mass spectra of each constituent with those stored in the Wiley/NIST 05.L database and with mass spectra from the literature (Adams 2009). The percentage composition of the oils was calculated in peak areas using normalization method.

### Statistical analysis

Hierarchical Cluster Analysis (HCA) and Principal Component Analyses (PCA) were used to evaluate whether the identified essential oils components can be useful for reflecting the chemotaxonomy of *P. mugo*. PCA and HCA analyses were performed using the statistical analysis software, XLSTAT Version 2014.2.03 (STATCON, Witzenhausen, Germany). The oil components with concentrations higher than 2 % (italic in Table 1) of the total oil were subjected to statistical analyses.

Hajdari Avni, Mustafa Behxhet, Ahmeti Gresa, Pulaj Bledar, Lukas Brigitte, Ibraliu Alban, Stefkov Gjoshe, Quave Cassandra L., Novak Johannes.
